# Short-term outcomes of intravitreal brolucizumab for treatment-naïve neovascular age-related macular degeneration with type 1 choroidal neovascularization including polypoidal choroidal vasculopathy

**DOI:** 10.1038/s41598-021-86014-7

**Published:** 2021-03-24

**Authors:** Hidetaka Matsumoto, Junki Hoshino, Ryo Mukai, Kosuke Nakamura, Hideo Akiyama

**Affiliations:** Department of Ophthalmology, Gunma University Graduate School of Medicine, 3-39-15 Showa-machi, Maebashi, Gunma 371-8511 Japan

**Keywords:** Medical research, Outcomes research

## Abstract

We evaluated the efficacy and safety of loading phase treatment with intravitreal brolucizumab for neovascular age-related macular degeneration (nAMD) with type 1 choroidal neovascularization (CNV). We analyzed consecutive 42 eyes of 40 patients with treatment-naïve nAMD associated with type 1 CNV. Three monthly injections of brolucizumab were completed in 36 eyes (85.7%). In those cases, best-corrected visual acuity (BCVA) was 0.24 ± 0.27 at baseline and improved significantly to 0.12 ± 0.23 after 3 months (*P* < 0.001). Central macular thickness was 301 ± 110 µm at baseline and decreased significantly to 160 ± 49 µm after 3 months (*P* < 0.001). Dry macula was achieved in 34 eyes (94.4%) after the loading phase. Central choroidal thickness was 264 ± 89 µm at baseline and decreased significantly to 223 ± 81 µm after 3 months (*P* < 0.001). Indocyanine green angiography after the loading phase revealed complete regression of polypoidal lesions in 15 of the 19 eyes (78.9%) with polypoidal lesions. Non-infectious intraocular inflammation (IOI) was observed in 8 of 42 eyes (19.0%) during the loading phase, while showing amelioration in response to combination therapy with topical and subtenon injection of steroids. In these eyes, BCVA after 3 months had not deteriorated as compared to that at baseline. These results indicate that loading phase treatment with intravitreal brolucizumab might be effective for improving visual acuity and reducing exudative changes in eyes with nAMD associated with type 1 CNV. Moreover, polypoidal lesions appear to frequently regress after this treatment. However, we must monitor patients carefully for brolucizumab-related IOI, and administer steroid therapy promptly.

## Introduction

Age-related macular degeneration (AMD) is a leading cause of visual impairment in developed countries^1^. Late AMD is divided into two subtypes: geographic atrophy and neovascular AMD (nAMD)^2^. nAMD is usually associated with choroidal neovascularization (CNV) and progresses more rapidly than geographic atrophy. CNV is divided into types 1 and 2 depending on its histopathological location^3^. Type 1 CNV grows beneath the retinal pigment epithelium (RPE). On the other hand, type 2 CNV grows into the subretinal space, thereby penetrating the RPE. Retinal angiomatous proliferation is a specific type of nAMD associated with intraretinal neovascularization, recently called type 3 neovascularization^4^. nAMD with type 1 CNV accompanied by polypoidal lesions is categorized as polypoidal choroidal vasculopathy (PCV)^5^. The prevalence of PCV in nAMD patients is higher in Asian than in Caucasian populations^6^. In eyes with PCV, massive submacular hemorrhage can occur due to bleeding from polypoidal lesions, leading to significant visual deterioration^7,8^.

Intravitreal injection of anti-vascular endothelial growth factor (VEGF) is the first line therapy for nAMD. Ranibizumab and aflibercept are anti-VEGF agents approved for ophthalmic use. In vitro studies have shown that aflibercept has higher anti-VEGF efficacy than ranibizumab^9^. Moreover, compared to ranibizumab, aflibercept is more effective for achieving regression of CNV beneath the RPE and polypoidal lesions as well as reducing choroidal thickness^10–12^. However, more than a few cases with type 1 CNV require frequent intravitreal injections of aflibercept to halt the development of CNV activity^13^.

Recently, brolucizumab was approved as a new anti-VEGF agent for the treatment of nAMD based on HAWK and HARRIER, worldwide phase 3 clinical trials^14,15^. These trials proved q12/q8 week dosing intervals for intravitreal brolucizumab to be effective for improving and maintaining visual acuity for 96 weeks, results not inferior to those of a q8 week dosing interval for intravitreal aflibercept. Moreover, intravitreal brolucizumab provided better control of intraretinal, subretinal, and sub-RPE fluid than intravitreal aflibercept. These results indicate that brolucizumab might be the most effective approach to treating nAMD with type 1 CNV among the anti-VEGF agents approved for ophthalmic use. Nevertheless, there are significant concerns about intraocular inflammation (IOI), including retinal occlusive vasculitis, after intravitreal brolucizumab which can result in permanent loss of vision^16^.

In this study, we evaluated efficacy and safety in the loading phase with intravitreal injections of brolucizumab for treatment-naïve nAMD with type 1 CNV.

## Results

**Figure 1:**
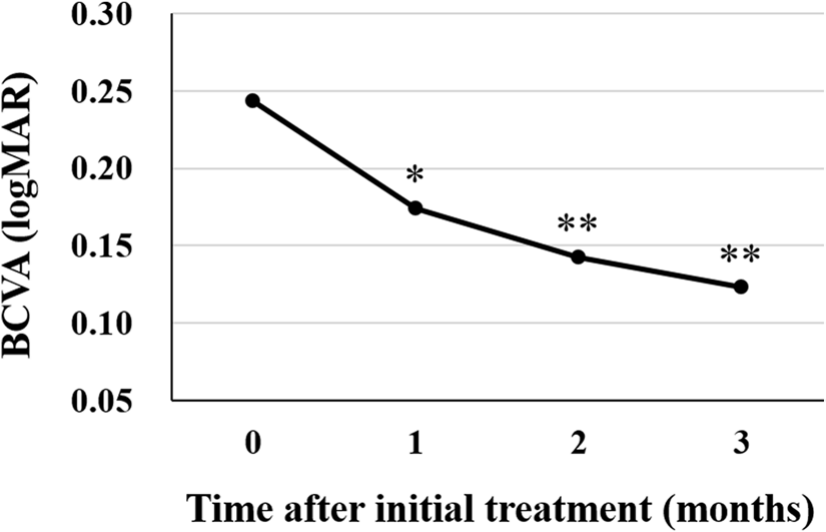
Change of average best-corrected visual acuity (BCVA) in 36 eyes with neovascular age-related macular degeneration associated with type 1 choroidal neovascularization treated with 3 monthly intravitreal injections of brolucizumab. BCVA showed significant improvement after the first injection of brolucizumab (*P < 0.01, **P < 0.001). Data are expressed as means.

**Figure 2:**
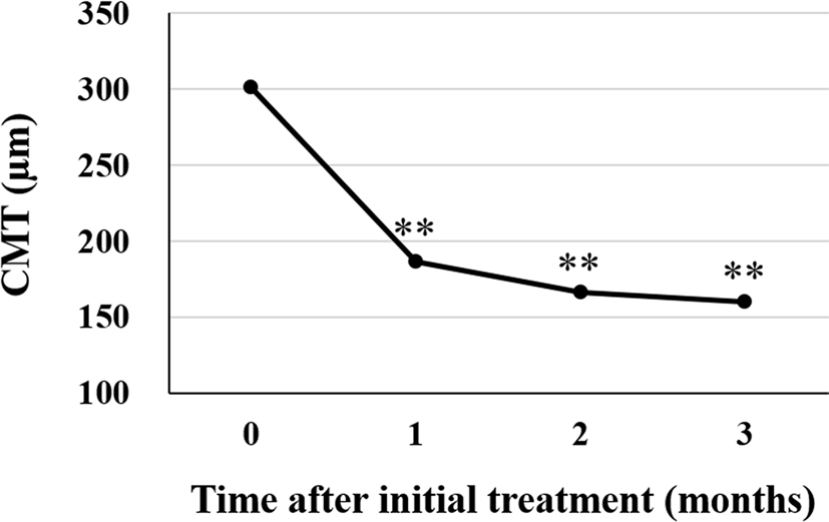
Change of average central macular thickness (CMT) in 36 eyes with neovascular age-related macular degeneration associated with type 1 choroidal neovascularization treated with 3 monthly intravitreal injections of brolucizumab. CMT showed significant improvement after the first injection of brolucizumab (**P < 0.001). Data are expressed as means.

**Figure 3:**
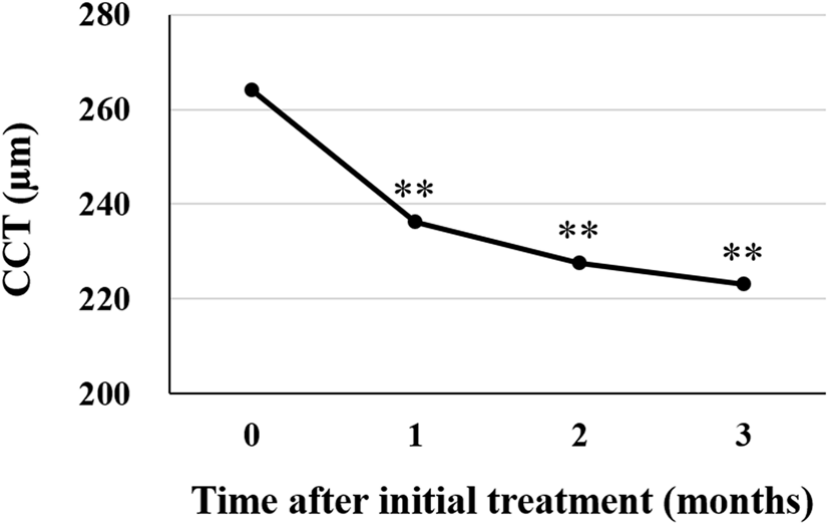
Change of average central choroidal thickness (CCT) in 36 eyes with neovascular AMD associated with type 1 choroidal neovascularization treated with 3 monthly intravitreal injections of brolucizumab. CCT showed significant improvement after the first injection of brolucizumab (**P < 0.001). Data are expressed as means.

**Figure 4:**
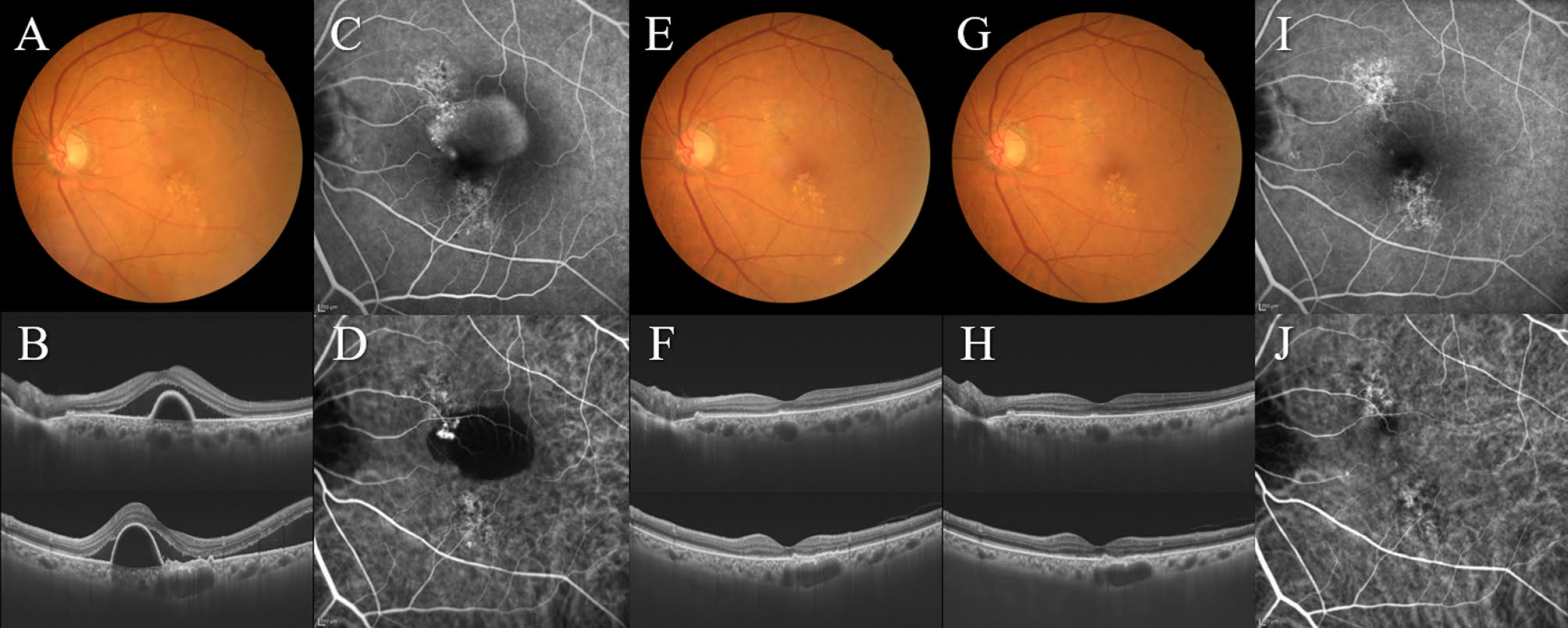
Images of the left eye of a 76-year-old man with polypoidal choroidal vasculopathy. Best-corrected visual acuity (BCVA) was 0.22 logarithm of the minimum angle of resolution (logMAR) units. (**A**) Color fundus photograph shows retinal pigment epithelium (RPE) degeneration and detachment accompanied by serous retinal detachment (SRD) at the macular area. (**B**) 12 mm horizontal and vertical B-mode optical coherence tomography (OCT) images through the fovea show dilated outer choroidal vessels associated with RPE detachment and SRD. The CMT and CCT are 388 μm and 360 μm, respectively. (**C**) Fluorescein angiography (FA) shows window defects, leakage, and pooling to the RPE detachment at the macular area. (**D**) Indocyanine green angiography (ICGA) shows polypoidal lesions in the RPE detachment. One month after the first injection of brolucizumab: BCVA of the left eye is 0.00 logMAR units. (**E**) Color fundus photograph shows neither RPE detachment nor SRD but RPE degeneration can be seen in the macular area. (**F**) 12 mm horizontal and vertical B-mode OCT images through the fovea show no SRD but there are dilated outer choroidal vessels associated with a shallow irregular RPE detachment. The CMT and CCT are 146 μm and 344 μm, respectively. One month after the third injection of brolucizumab: BCVA of the left eye is -0.08 logMAR units. (**G**) Color fundus photograph shows RPE degeneration at the macular area. (**H**) 12 mm horizontal and vertical B-mode OCT images through the fovea show dilated outer choroidal vessels associated with a shallow irregular RPE detachment. The CMT and CCT are 148 μm and 303 μm, respectively. (**I**) FA shows no leakage or pooling but there are window defects at the macular area. (**J**) ICGA shows no polypoidal lesions.

The subjects analyzed in this study included consecutive 42 eyes of 40 patients (35 eyes of 33 men; 7 eyes of 7 women) with treatment-naïve nAMD associated with type 1 CNV. Mean patient age was 74.9 ± 8.6 years. Twenty-three eyes (54.8%) showed type 1 CNV with polypoidal lesions. Three monthly injections of brolucizumab as a loading phase treatment were completed in 36 eyes (85.7%). In these cases, best-corrected visual acuity (BCVA) at baseline, after 1 month, 2 months, and 3 months were 0.24 ± 0.27, 0.17 ± 0.24 (*P* < 0.01), 0.14 ± 0.24 (*P* < 0.001), and 0.12 ± 0.23 (*P* < 0.001), respectively. BCVA showed significant improvement after the first injection of brolucizumab (Fig. 1). Central macular thickness (CMT) at baseline, after 1 month, 2 months, and 3 months were 301 ± 110 µm, 187 ± 71 µm (*P* < 0.001), 166 ± 53 µm (*P* < 0.001), and 160 ± 49 µm (*P* < 0.001), respectively. CMT was significantly decreased after the first intravitreal brolucizumab administration (Fig. 2). Dry macula was achieved in 17 eyes (47.2%) after 1 month, in 31 eyes (86.1%) after 2 months, and in 34 eyes (94.4%) after 3 months. Central choroidal thickness (CCT) at baseline, after 1 month, 2 months, and 3 months were 264 ± 89 µm, 236 ± 86 µm (*P* < 0.001), 227 ± 83 µm (*P* < 0.001), and 223 ± 81 µm (*P* < 0.001), respectively. CCT was significantly decreased after the first brolucizumab injection (Fig. 3). The 3 monthly injections of brolucizumab were completed in 19 of 23 eyes with polypoidal lesions; ICGA after the loading phase then revealed complete regression of the polypoidal lesions in 15 of these 19 eyes (78.9%). A representative case is shown in Fig. 4.

**Figure 5:**
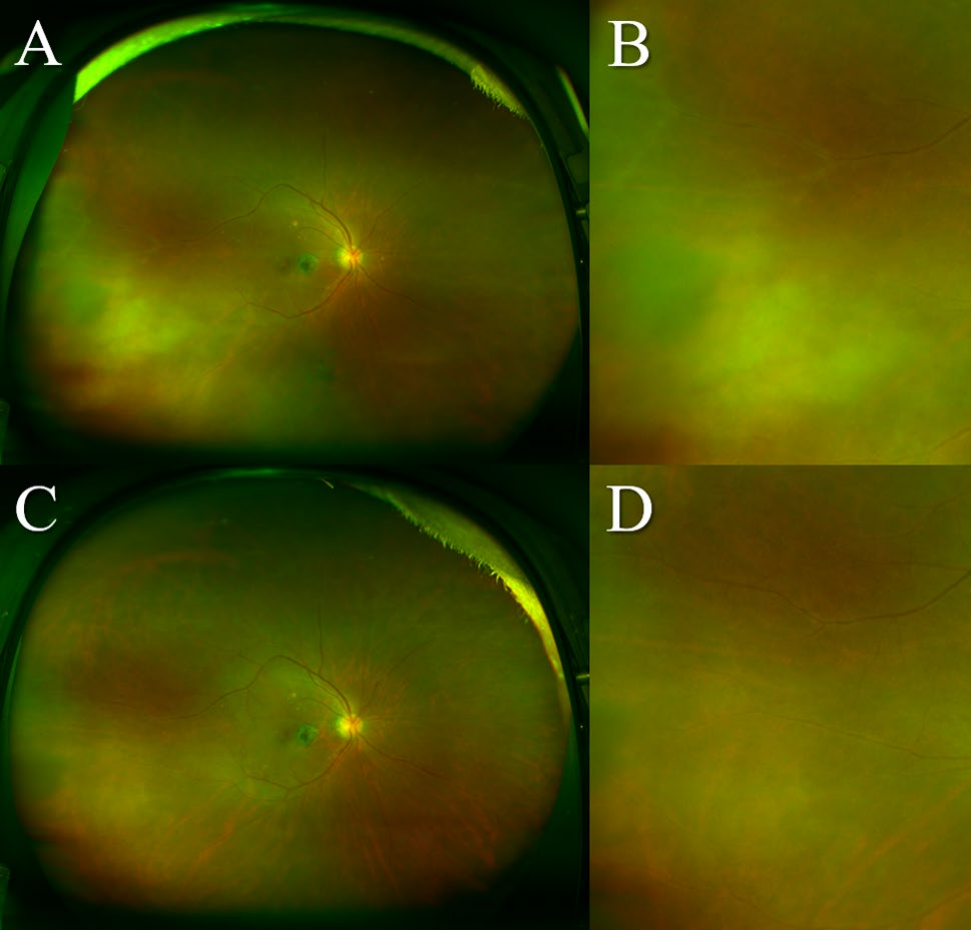
Fundus images of the right eye of a 77-year-old woman with polypoidal choroidal vasculopathy. She noticed floaters in her right eye approximately 3 weeks after the first injection of brolucizumab. Best-corrected visual acuity (BCVA) was 0.30 logarithm of the minimum angle of resolution (logMAR) units at 1 month after the first intravitreal brolucizumab injection. (**A**) Ultra-widefield color fundus imaging shows intraocular inflammation including vitritis and retinal vasculitis. (**B**) Magnified image of the temporal area of (**A**). Intravitreal brolucizumab therapy was stopped. She was promptly given combination therapy with subtenon injection of triamcinolone acetonide (30 mg/0.75 ml) and 0.1% betamethasone eye drops. BCVA of the right eye was improved to 0.10 logMAR units 1 month after the subtenon injection of triamcinolone acetonide. (**C**) Ultra-widefield color fundus imaging shows amelioration of the vitritis and retinal vasculitis. (**D**) Magnified image of the temporal area of (**C**).

Brolucizumab-related IOI was observed in 8 of 42 eyes (19.0%) during the loading phase; 3 eyes (37.5%) after the first injection of brolucizumab, 3 eyes (37.5%) after the second injection, and 2 eyes (25.0%) after the third injection. Iritis, vitritis, retinal vasculitis, retinal vascular occlusion, and papillitis were observed in 3 eyes (37.5%), 7 eyes (87.5%), 5 eyes (62.5%), 1 eye (12.5%), and 1 eye (12.5%), respectively. In these eyes, IOI was ameliorated by combination therapy with subtenon injection of triamcinolone acetonide (30 mg/0.75 ml) and 0.1% betamethasone eye drops (Fig. 5). Moreover, BCVA after 3 months had not deteriorated as compared to the baseline BCVA in any of the eyes with IOI. Demographic and clinical characteristics of cases with IOI after brolucizumab injection are presented in Table 1. When comparing baseline demographic and clinical characteristics between the cases with and without IOI detected during the loading phase, the cases with IOI were significantly older and more often female than those without IOI (*P* = 0.047 and 0.017, Table 2). No other severe adverse events, including infectious endophthalmitis, rhegmatogenous retinal detachment, cerebral infarction, and myocardial infarction, were observed in these patients.

**Table 1:** Demographic and clinical characteristics of cases with intraocular inflammation after brolucizumab injection.

Case	Age (years)	Gender	Period of onset (months)	Symptoms	Type of IOI					Treatment	Additional anti-VEGF after IOI	Status of macula at 3 months	BCVA at baseline	BCVA at 3 months
					Iritis	Vitritis	Retinal vasculitis	Retinal vascular occlusion	Papillitis					
1	76	M	0–1	None	〇					STTA (30 mg/0.75 ml) + 0.1% betamethasone eye drops	Aflibercept × 2	Dry	0.40	0.22
2	94	M	0–1	Floaters		〇				STTA (30 mg/0.75 ml) + 0.1% betamethasone eye drops	Aflibercept × 1	Dry	0.15	0.15
3	77	F	0–1	Floaters		〇	〇			STTA (30 mg/0.75 ml) + 0.1% betamethasone eye drops	None	Dry	0.15	0.10
4	73	M	1–2	Floaters		〇				STTA (30 mg/0.75 ml) + 0.1% betamethasone eye drops	None	Wet (sub-RPE fluid)	0.00	− 0.08
5	88	M	1–2	None		〇	〇	〇		STTA (30 mg/0.75 ml) + 0.1% betamethasone eye drops	None	Dry	0.30	0.10
6	78	F	1–2	Floaters		〇	〇			STTA (30 mg/0.75 ml) + 0.1% betamethasone eye drops	None	Wet (subretinal fluid)	0.22	0.00
7	72	F	2–3	Floaters	〇	〇	〇		〇	STTA (30 mg/0.75 ml) + 0.1% betamethasone eye drops	N/A	Dry	0.15	− 0.08
8	90	F	2–3	None	〇	〇	〇			STTA (30 mg/0.75 ml) + 0.1% betamethasone eye drops	N/A	Dry	0.05	− 0.08

*IOI* intraocular inflammation, *VEGF* vascular endothelial growth factor, *BCVA* best-corrected visual acuity, *STTA* subtenon injection of triamcinolone acetonide, *RPE* retinal pigment epithelium, *N/A* not available.

**Table 2:** Comparison of baseline demographic and clinical characteristics between cases with and without intraocular inflammation after brolucizumab injection.

	Total	IOI (+)	IOI (−)	*P* value
Number of eyes	42	8	34	
Age (years)	74.9 ± 8.6	81.0 ± 7.9	73.4 ± 8.1	0.047
Male	35 (83.3%)	4 (50.0%)	31 (91.2%)	0.017
Best-corrected visual acuity (logMAR)	0.24 ± 0.25	0.18 ± 0.12	0.25 ± 0.27	0.701
Central macular thickness (µm)	296 ± 109	275 ± 87	303 ± 110	0.543
Central choroidal thickness (µm)	264 ± 87	245 ± 75	269 ± 89	0.433
Polypoidal lesions (+)	23 (54.8%)	6 (75.0%)	17 (50.0%)	0.190

*IOI* intraocular inflammation.

## Discussion

We investigated outcomes in the loading phase of intravitreal brolucizumab for 42 eyes with treatment-naïve nAMD associated with type 1 CNV. In 36 eyes (85.7%) in which the 3 monthly injections of brolucizumab were completed, BCVA showed significant improvement, while CMT and CCT were significantly reduced during the loading phase. Moreover, polypoidal lesions showed complete regression in 15 of the 19 eyes (78.9%) with these lesions for which the treatment was completed. Brolucizumab-related IOI was observed in 8 of 42 eyes (19.0%) during the loading phase, but showed amelioration in response to combination therapy with topical and subtenon injection of steroids.

Massive submacular hemorrhage can occur in eyes with PCV due to the rupture of polypoidal lesions, leading to severe loss of vision^7,8^. Moreover, we previously reported that regression of polypoidal lesions after the loading phase allows fewer injections of aflibercept during the treatment of PCV utilizing a treat-and-extend regimen^17^. Therefore, regression of polypoidal lesions is one of the pivotal goals in treating PCV. The regression rate of polypoidal lesions after the loading phase with anti-VEGF agents was reported to be approximately 30% with the use of ranibizumab and about 50% with the use of aflibercept^17–22^. Photodynamic therapy combined with anti-VEGF injection reportedly achieved complete regression of polypoidal lesions in 70–90% of PCV patients 3 months after combination therapy^18–20,22,23^. In the current study, the regression rate after the loading phase with intravitreal brolucizumab was 78.9%, apparently higher than that achieved by monotherapy with ranibizumab or aflibercept, but similar to the regression rate observed after photodynamic therapy combined with anti-VEGF injection.

The decrease in subfoveal choroidal thickness after intravitreal aflibercept therapy was suggested to be related to visual gain and improvement of exudative changes in eyes with nAMD^24^. Previous studies revealed that the effect of reducing choroidal thickness is better with aflibercept than with ranibizumab^12^. The HAWK and HARRIER clinical trials have shown better control of sub-RPE fluid with brolucizumab than with aflibercept^14,15^. Therefore, it was speculated that brolucizumab reduces choroidal thickness more effectively than aflibercept, which might be related to better fluid control. In the current study, CCT was 264 ± 89 µm at baseline and 223 ± 81 µm after the loading phase. The CCT reduction rate in this study is slightly better than those obtained in previous studies using aflibercept^13,17^. Further studies are needed to elucidate the effect of reducing choroidal thickness with brolucizumab administration.

In this study, dry macula was achieved in 17 eyes (47.2%), 31 eyes (86.1%), and 34 eyes (94.4%) after the 1st, 2nd and 3rd of the monthly loading injections of brolucizumab, respectively. There results indicate the significant efficacy of fluid control achieved with brolucizumab. It is desirable to reduce the number of brolucizumab injections in order to reduce the risk of adverse events including retinal occlusive vasculitis leading to significant visual loss. Therefore, in the loading phase, monthly administration of brolucizumab for 3 months should perhaps not be recommended for all cases with nAMD.

Non-infectious IOI is one of the major adverse events encountered when administering intravitreal brolucizumab^16^. In the current study, 8 of 42 eyes (19.0%) showed IOI, including iritis, vitritis, retinal vasculitis, retinal vascular occlusion, and papillitis, during the loading phase with intravitreal brolucizumab. The IOI incidence was higher in our study than in the HAWK and HARRIER clinical trials^14–16^. Racial or ethnic differences might influence responses to brolucizumab^25^. In this study, the cases with IOI were significantly older and more often female than those without IOI, indicating that older and female patients might be more susceptible to brolucizumab-related IOI. Prior studies also showed that IOI following intravitreal brolucizumab affected females more than males^26,27^. In our study, IOI was observed after the second or third brolucizumab injection in 5 of 8 eyes with this adverse event, while there were no signs suggesting IOI after the first injection. Therefore, we provide a detailed explanation to all patients starting brolucizumab therapy, to insure that they understand the importance of seeking immediate ophthalmic examination whenever floaters or blurred vision are recognized after the injection. In terms of treating IOI after brolucizumab injection, this condition was ameliorated by steroid therapy in all of our cases. Early detection and treatment using steroids might be pivotal for managing brolucizumab-related IOI^28^. When the mechanisms underlying the development of IOI after brolucizumab injection are elucidated, it might be possible to predict and prevent this adverse event.

limitations of our study include the retrospective nature, single-center design, and small number of patients. All subjects were Japanese, and the results may therefore not be generalizable to nAMD in Caucasians and other racial or ethnic groups. Moreover, this study assessed only the loading phase of intravitreal brolucizumab therapy for nAMD with type 1 CNV. Thus, long-term outcomes and the efficacy of intravitreal brolucizumab for other nAMD subtypes still need to be evaluated.

In conclusion, loading phase treatment with intravitreal brolucizumab appeared to be effective for improving visual acuity and reducing exudative changes in eyes with nAMD associated with type 1 CNV. Moreover, polypoidal lesions appeared to frequently show regression after this treatment. However, it is essential to carefully monitor patients for brolucizumab-related IOI in order to avoid delaying the necessary steroid therapy.

## Methods

We performed this study, which complied with the guidelines of the Declaration of Helsinki, with approval from Institutional Review Board of Gunma University Hospital. Informed consent was obtained from all individual participants included in the study. We retrospectively studied consecutive 42 eyes of 40 patients with previously untreated nAMD associated with type 1 CNV. During the period from June 2020 through January 2021, the patients received 3 monthly intravitreal injections of brolucizumab as a loading phase at Gunma University Hospital.

All patients underwent a complete ophthalmic examination, including slit-lamp biomicroscopy with a noncontact fundus lens (SuperField lens; Volk Optical Inc, Mentor, OH), color fundus photography (Canon CX-1; Canon, Tokyo, Japan), ultra-widefield color fundus imaging (Optos 200Tx, Optos, Dunfermline, UK), fluorescein angiography (FA) and indocyanine green angiography (ICGA) (Spectralis HRA + OCT; Heidelberg Engineering, Heidelberg, Germany), as well as swept-source OCT (DRI OCT-1 Triton; Topcon Corp, Tokyo, Japan, and PLEX Elite 9000; Carl Zeiss Meditec, Dublin, CA, USA). In the OCT examination, we obtained B-mode images of the horizontal and vertical line scans (12 mm) through the fovea employing the DRI OCT-1 Triton. Then, we performed OCT angiography (OCTA) volume scanning, i.e., 300 × 300 pixels in the 3 × 3 mm area demonstrated by the PLEX Elite 9000. The OCTA thus performed was based on an optical microangiography algorithm. The diagnostic criteria for nAMD were based on a previous report^6^. We diagnosed nAMD with type 1 CNV if CNV was detected beneath the RPE by the aforementioned multimodal imaging. The presence of polypoidal lesions was evaluated on ICGA and B-mode OCT images, i.e., polyp-like choroidal vessel dilation on ICGA and sharply peaked RPE detachment on B-mode OCT^29^.

All eyes were treated by intravitreal injection of brolucizumab (6 mg/0.05 ml). When non-infectious IOI developed during the loading phase, brolucizumab therapy was stopped and subtenon injection of triamcinolone acetonide (30 mg/0.75 ml) as well as 0.1% betamethasone eye drops (3–6 times/day) were administered. All patients again underwent FA and ICGA 3 months after the first brolucizumab injection.

BCVA, CMT, and CCT were examined at every visit. BCVA was determined with manifest refraction and recorded as decimal values and converted to the logarithm of the minimal angle of resolution (logMAR) units. CMT and CCT were measured on B-scan OCT images using the computer-based caliper measurement tool in the OCT system. CMT was defined as the distance between the internal limiting membrane and the surface of the RPE at the fovea. CMT included any intraretinal and subretinal fluid. CCT was defined as the distance between Bruch’s membrane and the margin of the choroid and sclera under the fovea. Dry macula was defined as the macula without intraretinal, subretinal, and sub-RPE fluid accompanied by no or diminishing hemorrhage.

For statistical analyses, the Wilcoxon signed-rank test was used to compare the differences between BCVA, CMT and CCT at baseline versus other time points. The Fisher's exact test was used to examine the differences of gender and incidence of polypoidal lesions. Unpaired values of age, BCVA, CMT, and CCT were compared using the Mann–Whitney U test. The data analyses were performed using Excel 2016 (Microsoft, Redmond, WA, USA) with add-in software Statcel4^30^. A *P *<0.05 was considered to indicate a statistically significant difference. All data are presented as the average ± standard deviation.
